# Cross-species molecular mapping of the photoreceptor sensory cilium and periciliary complexes identifies conserved and species-specific architectural features

**DOI:** 10.64898/2026.05.24.727487

**Published:** 2026-05-27

**Authors:** Kei Takahashi, Mayuna Obayashi, Raghavi Sudharsan, William A. Beltran

**Affiliations:** 1 Division of Experimental Retinal Therapies, Department of Clinical Sciences & Advanced Medicine, School of Veterinary Medicine, University of Pennsylvania, Philadelphia, PA, USA.; 2 Biomedical Research Laboratory, Gifu Pharmaceutical University, Gifu, Japan.

**Keywords:** Calyceal processes, Canine, Human, Non-human primate, Photoreceptor sensory cilium, Ultrastructure expansion microscopy (U-ExM)

## Abstract

Photoreceptor sensory cilia (PSCs) support outer segment formation and are associated with specialized periciliary complexes, yet their cross-species and subtype-dependent organization remains incompletely defined. Here, we used ultrastructure expansion microscopy to compare PSC architecture and associated periciliary complexes across canine, non-human primate (NHP), and human retinas. Across all three species, rods and cones shared a conserved subtype-dependent organization, with cones displaying shorter connecting cilia (CCs), longer daughter centrioles (DCs), and enlarged bulge regions relative to rods. However, the scale and elaboration of these structures differed substantially between species, with primate PSCs showing shorter CCs, more pronounced widening of the apical region, and elongated cone DCs. Periciliary complexes showed even greater diversification. Canine rods exhibited a unilateral periciliary membrane and lacked calyceal process, whereas primate rods and cones of all three species showed a circumferential periciliary membrane and robust calyceal process. Human photoreceptors were broadly NHP-like but revealed variable accessory inner segment-like structures. Together, these findings provide a comparative nanoscale framework for photoreceptor ciliary organization in large mammals and show that conserved PSC principles coexist with pronounced species- and subtype-specific specialization.

## Introduction

Photoreceptors are light-sensing retinal neurons distinguished by a highly specialized ciliary compartment, known as the photoreceptor sensory cilium (PSC). In vertebrate retinas, photoreceptors are broadly classified into rods and cones, which are specialized for dim-light and daylight/color vision, respectively (Lamb, 2016; Molday and Moritz, 2015). The PSC comprises a tubulin-based structural core and the outer segment (OS), a highly specialized membrane organelle dedicated to phototransduction. The tubulin-based scaffold includes the daughter centriole (DC), basal body (BB), connecting cilium (CC), bulge region and distal axoneme, which together support the formation and maintenance of the OS. The OS begins to elaborate from the bulge region into stacked membrane lamellae or discs and requires a massive flux of membrane and protein trafficking for continual renewal (Barnes et al., 2021; Mercey et al., 2022).

In the periciliary region surrounding the base of the OS and the apical inner segment (IS), the PSC is accompanied by plasma membrane specializations, including a ciliary pocket and adjacent periciliary membrane domain that resemble corresponding subcompartments of conventional primary cilia (Sánchez-Bellver et al., 2021; Wensel et al., 2021). In many vertebrate photoreceptors, this region is further elaborated into a periciliary complex containing microvillus-like calyceal processes, which encircle the base of the OS. These structures are further distinguished by the compartmentalized distribution of Usher (USH) proteins, with USH2 proteins localized to the periciliary membrane region and USH1 proteins enriched at IS/OS boundary and calyceal process-associated membrane contacts (El-Amraoui and Petit, 2014; Sahly et al., 2012). Together, these highly specialized ciliary architectures, encompassing the PSC and its associated periciliary complexes, are thought to be broadly shared across photoreceptor subtypes and vertebrate species. However, detailed knowledge of their molecular organization and of the morphological features that distinguish photoreceptor subtypes and species remains limited.

Recent application of ultrastructure expansion microscopy (U-ExM) to the mature canine retina revealed striking differences between rod and cone photoreceptors across multiple PSC-associated subcompartments, including CC length, bulge region architecture, ciliary rootlet morphology and the organization of USH proteins (Takahashi et al., 2025). These findings established that subtype-specific molecular architecture can be resolved in formaldehyde-fixed frozen archival tissue of the adult large-animal retina at nanoscale resolution by utilizing U-ExM. However, whether such photoreceptor subtype-specific structural features are conserved across mammals, particularly in non-human primates (NHP), which are key translational models with close relevance to human photoreceptor biology, and in human photoreceptors themselves, has not been directly and systematically examined. Although the tubulin-based architecture of the PSC has not been systematically compared across species, previous work has shown that periciliary structures already exhibit pronounced interspecies and subtype-dependent variation: calyceal processes are well developed in NHP and human photoreceptors but absent or vestigial in rodents (Sahly et al., 2012; Verschueren et al., 2022). In addition, recent ultrastructural analysis identified a human rod-specific accessory inner segment, further supporting substantial species- and photoreceptor subtype-dependent specialization in structures surrounding the PSC (Lewis et al., 2025). Here, by applying U-ExM to formaldehyde-fixed frozen archival retinal samples from mature canine, non-human primate and human eyes, we compare PSC architecture and associated periciliary structures across photoreceptor subtypes and species, with the aim of refining current concepts of large-mammalian photoreceptor ciliary organization.

## Results

### Rod-cone differences in PSC architecture are conserved in dogs and NHPs but exhibit species-specific features

**Figure 1.**
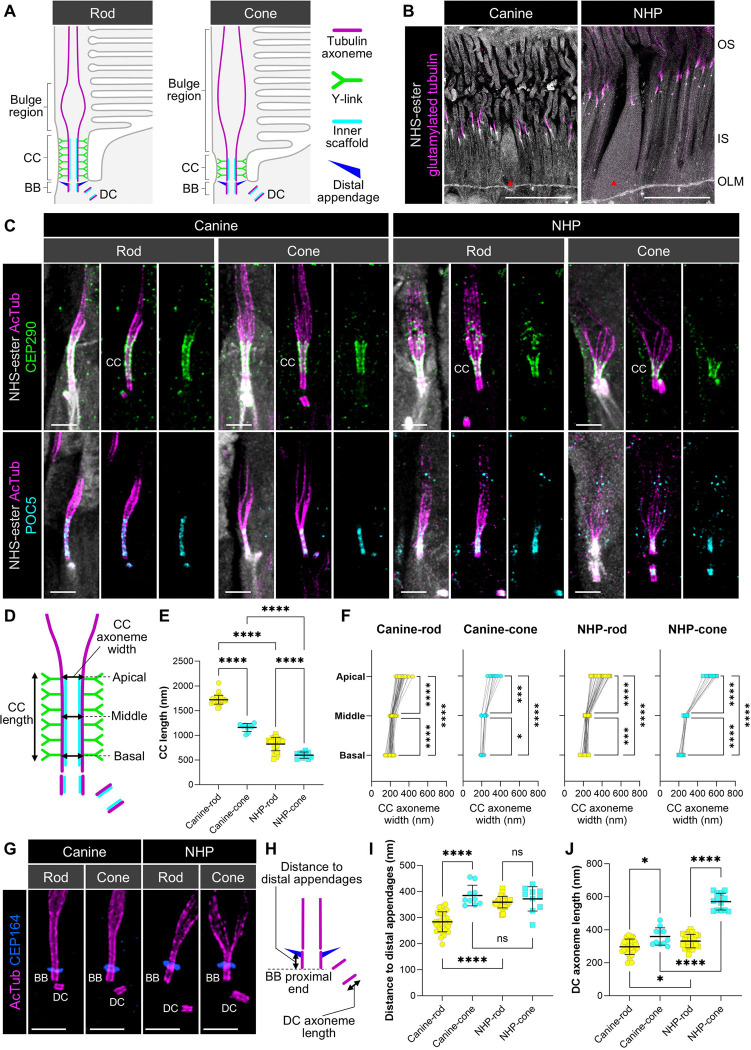
Cross-species analysis of the basal region of the PSC in canine and NHP photoreceptors. **(A)** Schematic diagram of rod (left) and cone (right) photoreceptor sensory cilia (PSCs). **(B)** Low-magnification confocal images showing an overview of the outer retina in canine (left) and non-human primate (NHP; right) eyes. Photoreceptors are outlined by N-hydroxysuccinimide (NHS)-ester pan-staining (gray). Overall, the NHP retina exhibits larger photoreceptor inner segments (IS) and outer segments (OS) than the canine retina. Tubulin-based PSC structures are visualized by glutamylation immunolabeling (magenta). All images are shown as maximum intensity projections (MIPs). Scale bars, 10 μm, corrected for the expansion factor. **(C)** Representative confocal images showing molecular localization in the connecting cilium (CC) of PSCs in rod and cone photoreceptors from canine and NHP retinas. Upper panels show CEP290 (green), a Y-link component of the CC, whereas lower panels show POC5 (cyan), a component of the tubulin inner scaffold. The PSC axoneme is visualized by acetylated α-tubulin (AcTub) immunolabeling (magenta). All images are shown as MIPs. Scale bars, 1 μm, corrected for the expansion factor. **(D)** Schematic diagram illustrating measurement of CC length and CC axoneme width. CC length was measured based on the CEP290-labeled Y-link distribution, whereas CC axoneme width was measured based on the AcTub-labeled axoneme at the corresponding region. **(E, F)** Quantification of CC length (E) and CC axoneme width (F) in canine and NHP retinas (canine, circles; NHP, squares). Central lines indicate the mean, and error bars represent ± SD. **(G)** Representative images highlighting the basal region of PSCs in canine and NHP photoreceptors. Distal appendages are visualized by CEP164 labeling (blue). All images are shown as MIPs. Scale bars, 1 μm, corrected for the expansion factor. **(H)** Schematic diagram illustrating measurement of the distance from the proximal end of the basal body (BB) to the distal appendages and of DC axoneme length. **(I, J)** Quantification of the distance to the distal appendages (I) and DC axoneme length (J) in canine and NHP retinas (canine, circles; NHP, squares). Central lines indicate the mean, and error bars represent ± SD. Data were obtained from three canine and two NHP eyes. *P < 0.05, ***P < 0.001, ****P < 0.0001, as assessed by Welch’s one-way ANOVA followed by Dunnett’s T3 multiple-comparison test.

In our recent study, we showed that the tubulin-based architecture of the young adult canine PSC differs markedly between rods and cones, with cones exhibiting shorter CCs, longer BBs and DCs, and a more extended bulge region than rods (Fig. 1A). To enable direct structural comparison between canine and NHP PSCs, we first established a common expansion factor (EF) using retinal samples that had undergone identical fixation and cryopreservation procedures. Following previous PSC studies using U-ExM (Faber et al., 2023; Mercey et al., 2024; Mercey et al., 2022), we estimated the EF from the width of the BB axoneme, based on the assumption that mother centriole width (231,3 nm) is conserved across mammalian species. BB axoneme width was comparable across species and photoreceptor subtypes, and an average EF of 3.64 was therefore used for all subsequent measurements (Supplementary Fig. S1).

To further minimize potential confounding factors in cross-species comparisons, we also considered whether PSC dimensions might vary according to retinal location. Because the canine values reported previously were derived from photoreceptors sampled across multiple retinal regions (Takahashi et al., 2025), we compared CC length between superior central and superior peripheral regions in the canine retina. CCs were modestly but significantly longer in both rods and cones in the superior central region than in the peripheral region (Supplementary Fig. S2). Therefore, to maintain regional consistency and avoid potential confounding factor from macular or foveal cone specialization, we used anatomically matched superior central retinal regions outside the macular/foveal region for all cross-species analyses described below.

Using N-hydroxysuccinimide (NHS)-ester counterstaining (M'Saad and Bewersdorf, 2020), which broadly labels cellular proteins, we were able to visualize the overall photoreceptor morphology and distinguish rods from cones based on their structural features without additional subtype-specific labeling. In low-magnification views, NHP retinas displayed markedly larger photoreceptor inner and outer segments than canine retinas in both rods and cones (Fig. 1B). This finding is consistent with the previous reports showing the larger primate photoreceptor IS/OS compared to rodents and other mammals, including dogs, based on conventional histological analysis (Booler et al., 2022; Grünert and Martin, 2020). In contrast, the overall dimensions of the tubulin-based PSC scaffold appeared broadly similar between canine and NHP photoreceptors (Fig. 1C), consistent with the comparable BB axoneme width observed across species and subtypes (Supplementary Fig. S1). Closer examination of individual PSC subcompartments, however, revealed clear subtype- and species-dependent differences.

We first focused on the CC, measuring its length based on centrosomal protein of 290 kDa (CEP290) signal distribution, a representative Y-link-associated marker of this compartment (Fig. 1D). As in canine retina, NHP cones exhibited significantly shorter CCs than NHP rods. In addition, CCs were significantly shorter in NHP than in canine photoreceptors in both subtypes (Fig. 1E). These subtype- and species-dependent differences in CC length were further supported by labeling with additional CC-associated markers, including spermatogenesis associated 7 (SPATA7) and glutamylation signals located outside the tubulin axoneme (Supplementary Fig. S3). By contrast, the width of the axoneme at the basal CC region was similar across species and photoreceptor subtypes and was consistent with the previously reported mouse CC axoneme width of approximately 200 nm (Gilliam et al., 2012) (Fig. 1F). However, axoneme width was not uniform along the CC and gradually increased toward the apical end in both species and subtypes. Notably, the apical widening of the CC was more prominent in NHP than in canine photoreceptors (Fig. 1F).

We then examined the BB and DC region in canine and NHP retinas (Fig. 1G, H). In canine cones, the distance from the proximal end of the BB to the centrosomal protein of 164 kDa (CEP164)-positive distal appendages was significantly greater than in canine rods, whereas no significant rod–cone difference was detected in NHP photoreceptors (Fig. 1I). In contrast, the feature of longer DCs in cones than in rods was shared between canine and NHP PSCs. Among all groups, NHP cones exhibited the longest DCs, approximately twice the length measured in the other groups (Fig. 1J).

**Figure 2.**
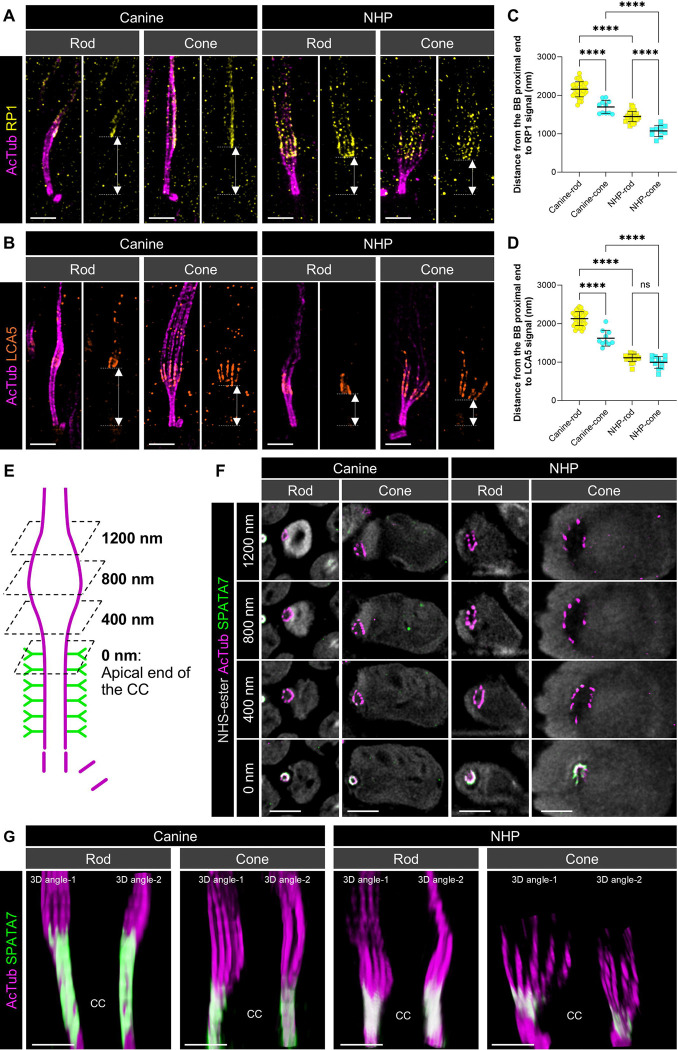
Organization of the apical region of the PSC in canine and NHP photoreceptors (A, B) Representative confocal images showing the molecular architecture of the apical region of PSCs in rod and cone photoreceptors from canine and NHP retinas. In addition to the AcTub-labeled axoneme (magenta), RP1 (yellow, A) and LCA5 (orange, B) were visualized by immunolabeling. White dashed lines and arrowheads indicate the distance from the proximal end of the BB to the RP1- or LCA5-positive region. All images are shown as MIPs. Scale bars, 1 μm, corrected for the expansion factor. (C, D) Quantification of the distance from the proximal end of the BB to RP1 (C) and LCA5 (D) signals in canine and NHP retinas (canine, circles; NHP, squares). Central lines indicate the mean, and error bars represent ± SD. Data were obtained from three canine and two NHP eyes. ****P < 0.0001, as assessed by Welch’s one-way ANOVA followed by Dunnett’s T3 multiple-comparison test. (E) Schematic diagram illustrating the imaging strategy for axial views of the apical region of PSCs. The distal end of the CC was used to define the 0 nm position. (F) Representative axial-view images of apical PSCs in canine and NHP retinas at 0, +400, +800, and +1200 nm from the distal end of the CC. The CC and axoneme were visualized by AcTub/SPATA7 immunolabeling (magenta/green), and the photoreceptor soma was visualized by NHS-ester pan-staining (gray). All images are shown as MIPs. Scale bars, 1 μm, corrected for the expansion factor. (G) Three-dimensional rendered images showing differences in bulge region width from the front (angle-1) and side (angle-2) views. Scale bars, 1 μm, corrected for the expansion factor.

To define the structural organization of the apical PSC region, we next labeled RP1 and LCA5, molecular markers of the distal axoneme and bulge region, respectively (Fig. 2A, B). The distances from the proximal end of the BB to retinitis pigmentosa 1 protein (RP1) and Leber congenital amaurosis 5 protein (LCA5) signals reflected the differences in CC length across photoreceptor subtypes and species, with canine rods showing the longest and NHP cones the shortest values (Fig. 2C, D). In addition, the distribution patterns of RP1 and LCA5 indicated that NHP photoreceptors possess a longer and wider bulge region than canine photoreceptors. To examine this region in more detail, we performed axial-view imaging of the distal PSC (Fig. 2E, F). In canine rods, widening of the tubulin axoneme was evident at +400 and +800 nm from the distal end of the CC, but had already begun to taper by +1200 nm. By contrast, canine cones showed sustained axonemal expansion even at +1200 nm. (Fig. 2F). In NHP photoreceptors, both rods and cones displayed bulge regions that were wider and longer than those in canine rods, with NHP cones showing the most pronounced expansion among all groups. In addition, the bulge region in NHP photoreceptors exhibited a more elliptical morphology than in canine photoreceptors (Fig. 2F). These species- and subtype-dependent structural differences were further supported by three-dimensional reconstructions (Fig. 2G).

Taken together, the fundamental rod–cone differences in tubulin-based PSC architecture were broadly conserved between canine and NHP photoreceptors: cones possessed shorter CCs, longer DCs, and a larger bulge region than rods. At the same time, clear species-specific differences were also evident, particularly in CC length, the extent of apical CC widening, and the morphology of the bulge region.

### Human PSC architecture aligns with primate features while retaining rod-cone differences

**Figure 3.**
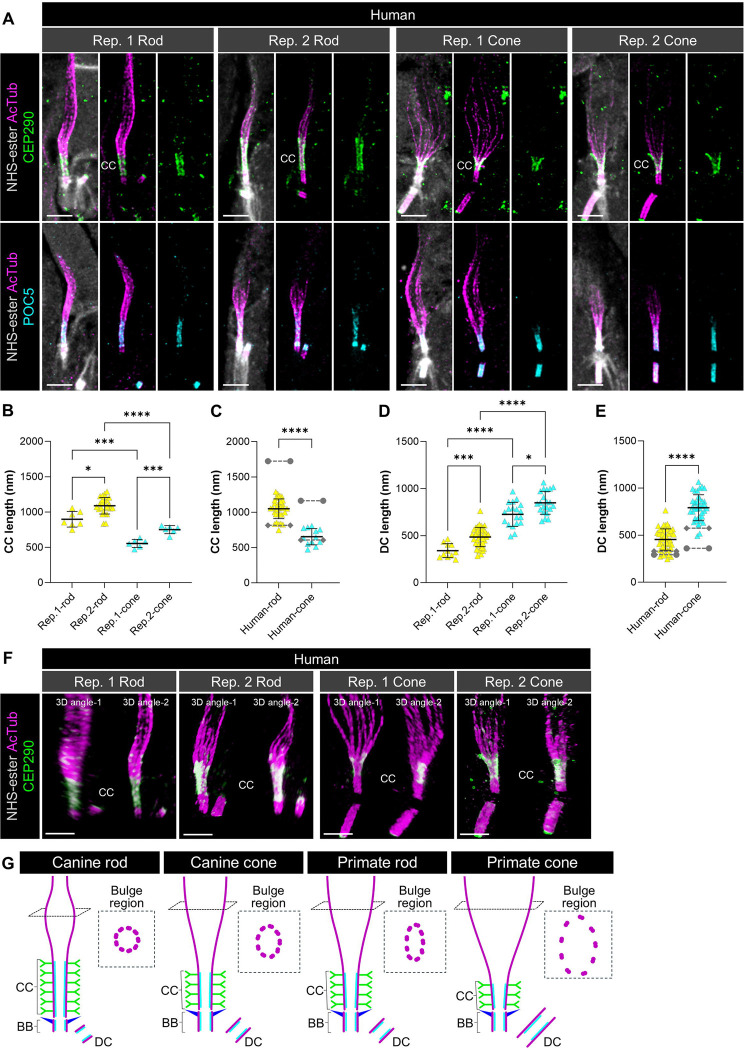
Molecular architecture of human PSCs in the context of canine and NHP PSC features (A) Representative confocal images showing the tubulin axoneme visualized by AcTub immunolabeling (magenta) together with CEP290 (upper, green) and POC5 (lower, cyan) localization in human rod and cone photoreceptors from two independent biological replicates. All images are shown as MIPs. Scale bars, 1 μm, corrected for the expansion factor. (B-E) Quantification of CC length (B, C) and DC axoneme length (D, E). Graphs B and D show values for individual biological replicates, whereas C and E show the combined results. Gray dashed lines indicate the mean values for canine and NHP photoreceptors of each subtype, based on the corresponding measurements shown in Fig. 1E, J (canine, circles; NHP, squares). Central lines indicate the mean, and error bars represent ± SD. Data were obtained from two human eyes. *P < 0.05, ***P < 0.001, ****P < 0.0001, as assessed by Welch’s one-way ANOVA followed by Dunnett’s T3 multiple-comparison test (B, D) and Welch’s *t*-tests (C, E). (F) Three-dimensional rendered images showing differences in bulge region width from the front (angle 1) and side (angle 2) views. (G) Schematic summary diagrams illustrating structural differences between rod and cone PSCs across canine and primate retinas. Dashed squares indicate axial views of tubulin axoneme organization in the bulge region.

Having established both shared and species-specific features of PSC architecture between canine and NHP photoreceptors, we next extended the comparison to human photoreceptors. Unlike the canine and NHP samples, which were obtained from young adult animals processed under standardized conditions, the human donor retinas were derived from elderly donors (>80 years old) and showed substantial variation in postmortem interval and fixation conditions (Supplementary Table S1). U-ExM analysis revealed noticeable variation in PSC morphology between the two human biological replicates (Fig. 3A). The sources of this variability remain unclear and may reflect differences in sample conditions and/or donor-specific factors. We therefore analyzed the human data both by combining measurements from the two donor eyes and by examining each biological replicate separately.

Despite the inter-replicate variability, several fundamental subtype-specific features were preserved. For CC length, significant differences were observed between the two human biological replicates, but in both samples cone CCs remained shorter than rod CCs, consistent with the findings in canine and NHP retinas (Fig. 3B). When the combined human data were compared with the cross-species averages, both rod and cone CCs were shorter than those in canine photoreceptors and more closely resembled those in NHP photoreceptors (Fig. 3C). A similar pattern was observed for DC length. Although DC length also differed significantly between the two human biological replicates, cones consistently exhibited longer DCs than rods, again in agreement with the subtype-specific pattern shared by canine and NHP photoreceptors (Fig. 3D). Notably, whereas NHP cones already displayed exceptionally long DCs, human cones exhibited even greater DC elongation than NHP cones in the combined analysis (Fig. 3E).

Given the striking elongation of the DCs in NHP and human cones, we next asked whether this feature differed between cone subtypes. Using glutamylation labeling together with cone subtype-specific opsin labeling, we measured CC and DC length in S-cones and L/M-cones across the three species (Supplementary Fig. S4A). CC length did not significantly differ between cone subtypes in canine, NHP or human retinas (Supplementary Fig. S4B). In contrast, DC length was consistently greater in L/M-cones than in S-cones in all three species (Supplementary Fig. S4C).

The apical PSC region in human photoreceptors also resembled that of NHPs more closely than that of canines. In both rods and cones, the bulge region was more extended than in canine photoreceptors, and this was particularly pronounced in cones, which showed a greater expansion of the axoneme similar to that observed in NHP cones (Fig. 3F). Together, these findings indicate that the molecular architecture of tubulin-based PSC axoneme differs between canine and primate photoreceptors, whereas human and NHP photoreceptors share many common structural features. At the same time, the fundamental rod–cone differences in PSC architecture were maintained across all species, with cones consistently exhibiting shorter CCs, longer DCs and a larger bulge region than rods (Fig. 3G).

### Periciliary complexes exhibit photoreceptor subtype- and species-dependent organization in canine and NHP retinas

**Figure 4.**
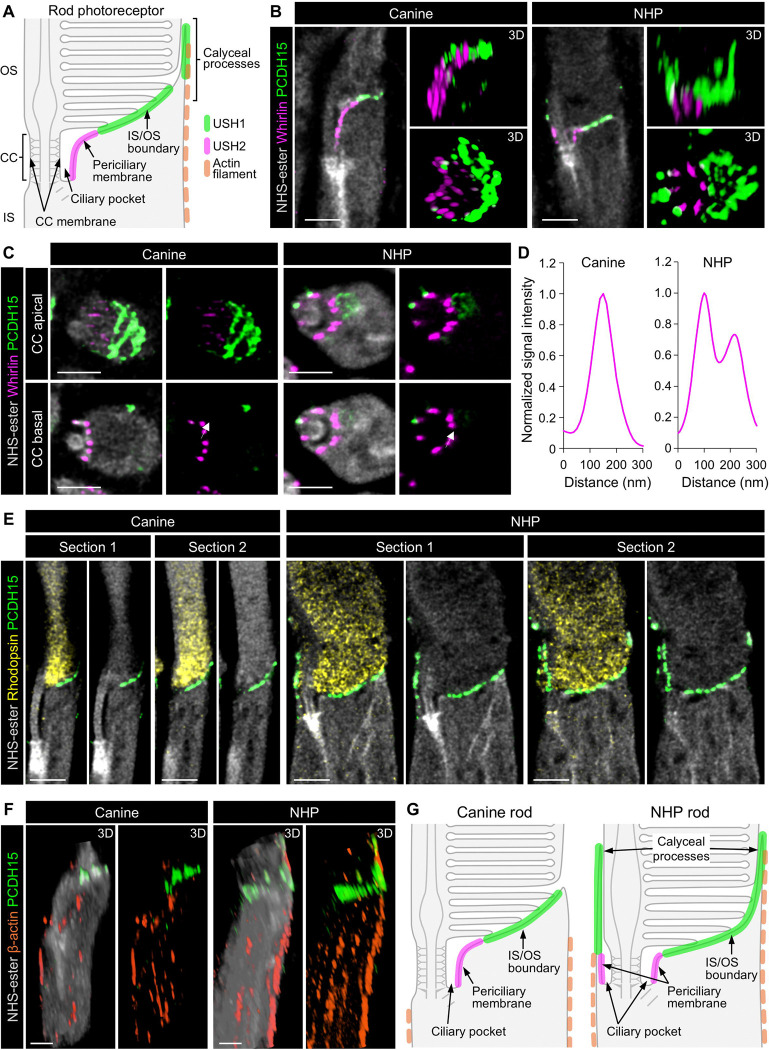
Periciliary complex organization in canine and NHP rod photoreceptors (A) Schematic diagram of periciliary structures in rod PSCs based on previous publications (Sahly et al., 2012). The USH1- and USH2-associated domains shown here summarize representative localization patterns reported for selected Usher protein groups at the IS/OS boundary, calyceal process, and periciliary membrane regions, and are not intended to represent all reported subcellular localizations of individual USH proteins. (B) Representative images showing molecular localization in periciliary structures of rod PSCs from canine and NHP retinas. Representative Usher proteins were visualized by immunolabeling for PCDH15 (USH1F, green) and Whirlin (USH2D, magenta), together with NHS-ester pan-staining (gray). For both species, left panels show single optical sections, whereas right panels show three-dimensional rendered images in lateral (upper) and axial (lower) views. Scale bars, 1 μm, corrected for the expansion factor. (C) Axial views of periciliary structures in rod photoreceptors corresponding to the basal (lower) and apical (upper) regions of the CC. All images are shown as single optical sections. Scale bars, 1 μm, corrected for the expansion factor. (D) Normalized line intensity profiles of Whirlin distribution along the periciliary membrane, corresponding to the white arrows indicated in (C). X-axis values are corrected for the expansion factor. (E) Single optical sections showing the distribution pattern of PCDH15 in periciliary structures of canine and NHP rods. The OS region is visualized by rhodopsin immunolabeling (yellow). For both canine and NHP images, section 1 corresponds to the region containing the CC, whereas section 2 shows the mid-peripheral region. Scale bars, 1 μm, corrected for the expansion factor. (F) Three-dimensional rendered images showing the distribution pattern of actin filaments associated with calyceal processes. Actin filaments are visualized by β-actin immunolabeling (orange). Scale bars, 1 μm, corrected for the expansion factor. (G) Schematic diagram illustrating the updated structural characteristics of periciliary structures in canine and NHP rod photoreceptors.

Previous work in primate photoreceptors has shown that the periciliary complexes contain calyceal processes and distinct Usher protein domains, with USH2 proteins localized to the periciliary membrane region and representative USH1 proteins associated with the IS/OS boundary and calyceal process-related membranes (Sahly et al., 2012) (Fig. 4A). By contrast, calyceal processes are absent or vestigial in rodents, highlighting marked interspecies differences in this compartment. Because the precise structural boundaries and nanoscale molecular organization of these periciliary complexes remain incompletely defined, we next compared their organization between canine and NHP photoreceptors following the framework outlined above. To visualize representative components of USH1 and USH2 protein complexes, we performed immunolabeling for protocadherin-15 (PCDH15) and Whirlin, respectively, in combination with NHS-ester counterstaining, which enabled clear delineation of periciliary subcompartments (Fig. 4B).

In both canine and NHP rods, Whirlin localized to the periciliary membrane domain, consistent with previous reports. However, pronounced species-dependent differences in its spatial distribution were evident. In lateral views, Whirlin signal in canine rods was restricted to the region between the CC and the IS, whereas in NHP rods it was detected on both sides of the CC. Three-dimensional reconstructions further revealed that Whirlin in canine rods covered only a semicircular domain corresponding to the IS-facing side of the CC, whereas in NHP rods Whirlin-positive structures formed six pillar-like elements that surrounded the entire circumference of the CC (Fig. 4B). To further resolve this distribution pattern, we performed higher-magnification axial imaging. NHS-ester counterstaining revealed that the periciliary membrane in canine rods was confined to the IS side of the CC and was absent on the opposite side, whereas in NHP rods it extended around the full circumference of the CC. The Whirlin-positive pillar-like structures were localized along this periciliary membrane domain (Fig. 4C). Notably, these pillar-like structures also differed in their organization: Whirlin-positive elements in canine rods appeared as approximately six to seven discrete singlets, whereas in NHP rods they were arranged as six pairs of adjacent pillar-like structures around the CC (Fig. 4D).

Similarly, the distribution of the USH1 protein PCDH15 also showed clear species-dependent differences. In canine rods, PCDH15 signal was largely restricted to the IS/OS boundary region. In NHP rods, however, PCDH15 was detected not only at the IS/OS boundary but also as axially oriented signals extending along the basal OS (Fig. 4B, E). Because calyceal processes are known to be associated with actin filaments extending from the IS, we next examined the organization of β-actin in this region. In canine rods, actin filaments within the IS appeared discontinuous and lacked clear association with PCDH15-positive structures, consistent with our previous observations (Takahashi et al., 2025). In contrast, NHP rods exhibited prominent, continuous actin filaments that were closely aligned with PCDH15 signals, forming robust filamentous profiles extending from the IS toward the OS base (Fig. 4F).

Together, these observations reveal marked species-dependent differences in the organization of rod periciliary complexes. In canine rods, the periciliary membrane and ciliary pocket were restricted to the IS-facing side of the CC, whereas in NHP rods these structures extended circumferentially around the CC. Furthermore, the distribution patterns of PCDH15 and associated actin filaments suggest that calyceal processes are absent or only weakly developed in canine rods, whereas NHP rods display clear molecular features consistent with well-defined calyceal processes (Fig. 4G).

**Figure 5.**
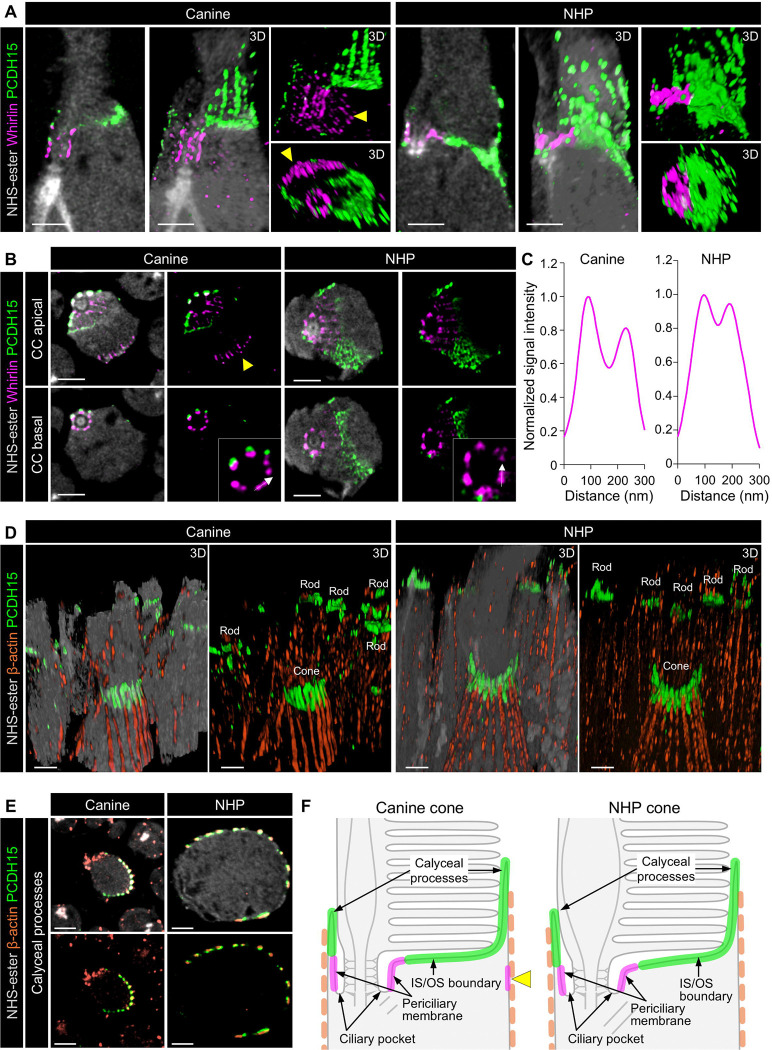
Molecular organization of periciliary complexes in canine and NHP cone photoreceptors (A) Representative images showing molecular localization in periciliary structures of cone PSCs from canine and NHP retinas. PCDH15 (USH1F, green) and Whirlin (USH2D, magenta) were visualized by immunolabeling, together with NHS-ester pan-staining (gray). For both species, left panels show single optical sections, whereas middle and right panels show three-dimensional rendered images in lateral and axial views. Yellow arrowheads indicate ectopic Whirlin localization in canine cones. Scale bars, 1 μm, corrected for the expansion factor. (B) Axial views of periciliary structures in cone photoreceptors at the basal (lower) and apical (upper) regions of the CC. Yellow arrowhead indicates ectopic Whirlin localization in canine cones. All images are shown as single optical sections. Scale bars, 1 μm, corrected for the expansion factor. (C) Normalized line intensity profiles of Whirlin distribution along the periciliary membrane, corresponding to the white arrows indicated in (B). X-axis values are corrected for the expansion factor. (D) Three-dimensional rendered images showing the distribution pattern of actin filaments associated with calyceal processes. Actin filaments are visualized by β-actin immunolabeling (orange). Scale bars, 1 μm, corrected for the expansion factor. (E) Axial views of calyceal processes in canine (left) and NHP (right) cone photoreceptors. All images are shown as single optical sections. Scale bars, 1 μm, corrected for the expansion factor. (F) Schematic diagram illustrating the updated structural characteristics of periciliary structures in canine and NHP cone photoreceptors.

Using the same approach, we next compared the organization of periciliary complexes in cone photoreceptors from canine and NHP retinas. In contrast to rods, lateral views showed Whirlin-positive pillar-like structures on both sides of the CC in cones of both species. Three-dimensional reconstructions further revealed circumferential arrays of Whirlin-positive structures surrounding the CC in both canine and NHP cones, resembling the pattern observed in NHP rods (Fig. 5A). Notably, however, three-dimensional rendering also revealed an additional ectopic Whirlin-positive cluster in canine cones that was not associated with the periciliary membrane and was not observed in any other photoreceptor subtype (Fig. 5A). This ectopic Whirlin signal was consistently detected in all canine biological replicates examined, although its precise distribution pattern varied between cells (Supplementary Fig. S5). High-magnification axial imaging further showed that, in both canine and NHP cones, the Whirlin-positive pillar-like structures were organized as paired doublet-like elements distributed circumferentially around the CC, similar to those observed in NHP rods (Fig. 5B, C). However, the number of these paired structures differed between species: canine cones exhibited five pairs, whereas NHP cones displayed six pairs, matching the number observed in NHP rods (Fig. 5B). The ectopic Whirlin-positive cluster unique to canine cones was localized along the IS plasma membrane (Fig. 5B).

PCDH15 exhibited a broadly similar distribution pattern in cones of both species. In canine and NHP cones, PCDH15 was detected not only at the IS/OS boundary but also as axially oriented signals extending along the basal part of the OS (Fig. 5A). These PCDH15-positive profiles were clearly associated with actin filaments extending from the IS, supporting the presence of calyceal process in cones of both species (Fig. 5D, E).

Together, these findings indicate that cone photoreceptors in both canine and NHP retinas possess circumferential ciliary pocket/periciliary membrane organization around the CC, accompanied by clearly defined calyceal processes. At the same time, species-dependent differences were also evident, including the number of Whirlin-positive paired pillar-like structures and the presence of an ectopic Whirlin-positive domain uniquely observed in canine cones (Fig. 5F).

### Human periciliary complexes are broadly primate-like but also show species-dependent features

**Figure 6.**
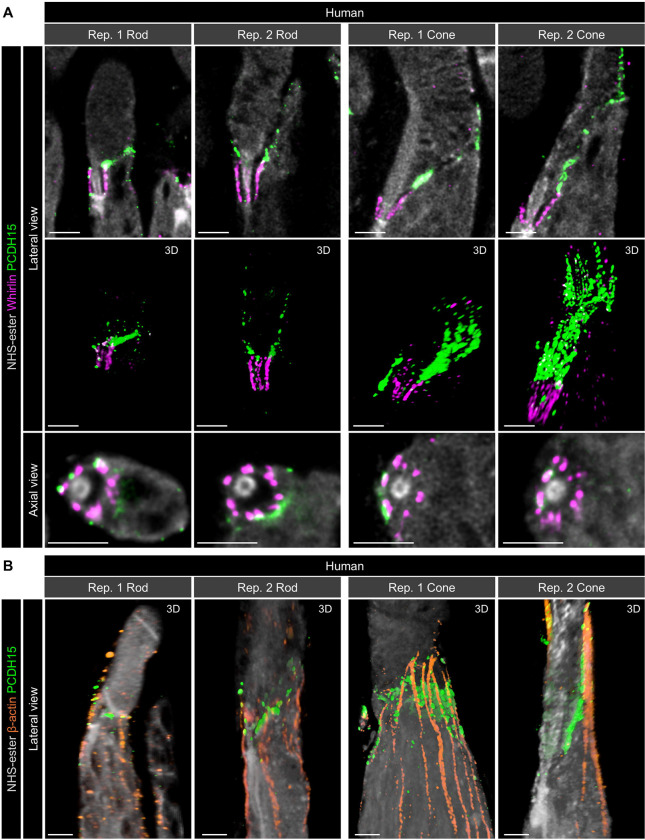
Periciliary complex architecture in human photoreceptors (A) Representative images showing molecular localization in periciliary complex of rod and cone PSCs in human retina from two individual biological replicates. PCDH15 (USH1F, green) and Whirlin (USH2D, magenta) were visualized by immunolabeling, together with NHS-ester pan-staining (gray). Upper panels show single optical sections in lateral views, middle panels show three-dimensional rendered images in lateral views, and lower panels highlight axial view images at the regions of the CC. Scale bars, 1 μm, corrected for the expansion factor. (B) Three-dimensional rendered images showing the distribution pattern of actin filaments associated with calyceal processes. Actin filaments are visualized by β-actin immunolabeling (orange). Scale bars, 1 μm, corrected for the expansion factor.

We next extended the analysis of periciliary complexes to human photoreceptors. In both biological replicates, rod and cone photoreceptors exhibited six pairs of Whirlin-positive pillar-like structures arranged circumferentially around the CC (Fig. 6A), consistent with the organization observed in NHP photoreceptors. In addition, both rods and cones showed PCDH15 signal not only at the IS/OS boundary but also as axially oriented profiles extending along the basal OS. Actin filaments extending toward these PCDH15-positive structures were also evident, further supporting a NHP-like periciliary organization in human photoreceptors (Fig. 6A).

Notably, the calyceal process-like structures delineated by PCDH15 and β-actin were more robust in cones than in rods (Fig. 6B). This subtype-dependent difference was consistent with the pattern observed in NHP photoreceptors and with previous reports describing more robust calyceal process organization in primate cones than in rods (Sahly et al., 2012; Verschueren et al., 2022). Together, these findings indicate that human periciliary complexes broadly resemble those of NHP photoreceptors while retaining clear rod–cone differences in their structural organization.

Beyond these broadly primate-like periciliary features, we next asked whether human photoreceptors also display additional PSC-associated structures not observed in canine or NHP retinas. A recent electron microscopy-based ultrastructural study described a previously unrecognized structure unique to human rods, termed the accessory inner segment (aIS), in which the IS extends alongside the OS and is reinforced by a prominent microtubule-based scaffold. This structure was further reported to contain mitochondria extending into the OS region and to be absent from human cones (Lewis et al., 2025). Because this finding is highly recent and has not yet been systematically evaluated in other species or by alternative imaging approaches, we used U-ExM to assess the presence and structural characteristics of aIS-like structures in canine, NHP, and human photoreceptors.

**Figure 7.**
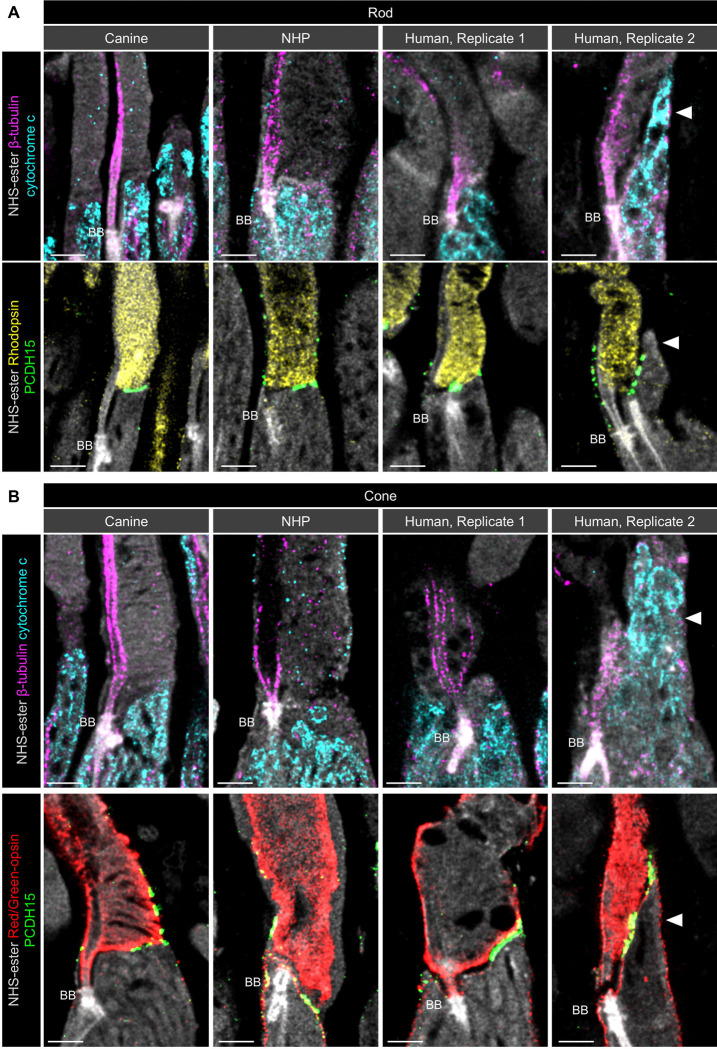
Presence or absence of the accessory inner segment across three mammalian species (A) Representative single optical section images of rod photoreceptors showing the distribution of inner segment (IS) mitochondria (upper panels, cyan) and the IS/OS boundary visualized by rhodopsin/PCDH15 immunolabeling (lower panels, yellow/green). Only in human replicate 2, several rod photoreceptors showed infiltration of mitochondria into the OS region and protrusion of the IS soma into the OS region, indicating the presence of an accessory inner segment (aIS; white arrowheads). In rod photoreceptors from canine, NHP, and human replicate 1 retinas, these structural features were not observed in any cells. (B) Representative images of cone photoreceptors showing the distribution of IS mitochondria (upper panels, cyan) and the IS/OS boundary visualized by L/M-opsin/PCDH15 immunolabeling (lower panels, red/green). Similar to the observations in rod photoreceptors, mitochondrial infiltration into the OS region was observed in several cone photoreceptors only in human replicate 2, indicating that the aIS is also present in cone photoreceptors. All images are shown as single optical sections. Scale bars, 1 μm, corrected for the expansion factor.

We first examined rod photoreceptors, in which the aIS has been proposed to be a human-specific feature (Lewis et al., 2025). In canine and NHP rods, we found no structural features suggestive of an aIS. Consistent with a conventional rod morphology, cytochrome c-labeled mitochondria were clearly confined to conventional IS region in both species (Fig. 7A). Human samples, however, showed distinct findings between biological replicates. One donor retina (replicate 1) displayed rod morphology comparable to that of canine and NHP rods, whereas the other (replicate 2) contained rod profiles with aIS-like structures extending alongside the outer segment. In these cells, cytochrome c-positive mitochondria were also detected within the extended structure, consistent with one of the defining features of the aIS described previously. The extent of this aIS-like structure varied markedly among rods. In contrast to the prior electron microscopy study, we did not detect an additional tubulin-based scaffold located on the opposite side of the IS relative to the ciliary axoneme, which has been proposed to provide structural support to the aIS (Lewis et al., 2025). Consistent with the interpretation that this structure represented an IS-derived extension running alongside the OS, PCDH15 signal marked the boundary between the OS and the aIS-like structure (Fig. 7A).

We next performed the same analysis in cone photoreceptors. As in rods, no comparable structure was observed in canine or NHP cones. In the human samples, replicate 1 again lacked any prominent abnormality, whereas replicate 2 contained cones with IS-derived structures extending alongside the OS (Fig. 7B). As in rods, these structures were confirmed by morphological features visualized by NHS-ester staining, by the presence of mitochondria in the OS-associated region, and by an abnormally elongated PCDH15-positive IS/OS boundary (Fig. 7B). Thus, unlike the previous report describing the aIS as a rod-specific feature of human photoreceptors, our observations suggest that aIS-like structures may also occur in human cones under at least some conditions.

Together, these findings support the possibility that aIS-like structures are a distinctive feature of human photoreceptors not observed in canine or NHP retinas. At the same time, our data indicate that their structural and molecular characteristics, their occurrence in rod versus cone photoreceptors, and their consistency across human donor samples remain unresolved. These observations therefore extend the recent ultrastructural description of the human aIS while also raising questions about its generality and subtype specificity.

### Species- and subtype-dependent diversity extends to ciliary rootlet associated with the PSC

**Figure 8.**
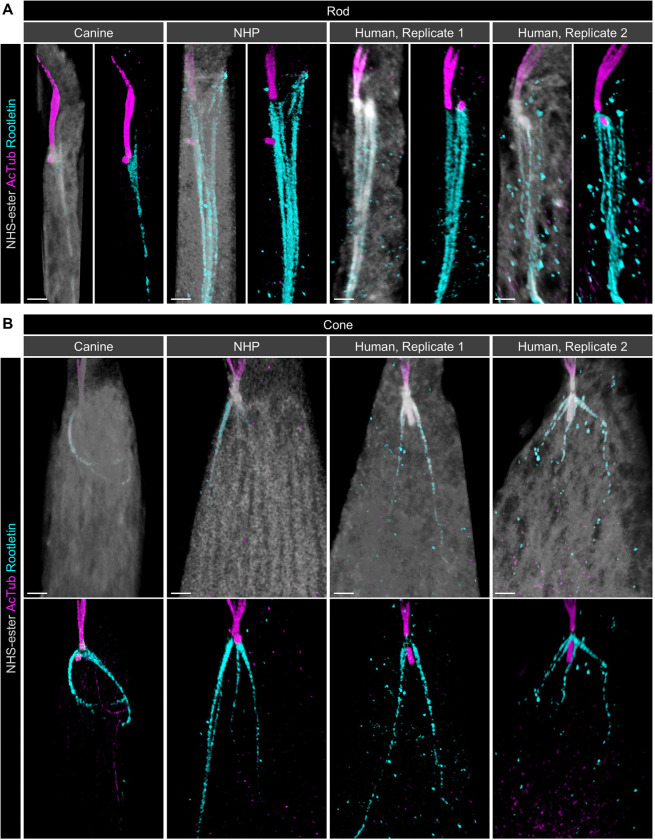
Cross-species comparison of ciliary rootlet architecture in PSCs from three mammals (A, B) Representative images of rod (A) and cone (B) photoreceptors immunolabeled for AcTub (magenta) and rootletin (cyan), together with NHS-ester pan-staining (gray). NHP and human rod photoreceptors possess thicker rootlets than canine rod photoreceptors (A). Whereas the rootlet in canine cones exhibits a curvilinear morphology, NHP and human cones display rootlets that branch into two or three filaments (B). All images are shown as three-dimensional rendered images. Scale bars, 1 μm, corrected for the expansion factor.

Finally, we assessed the morphological characteristics of the ciliary rootlet in photoreceptors across the three species. In our previous study of canine retina, we found that ciliary rootlet morphology differs strikingly between rod and cone photoreceptors. Specifically, canine rods exhibited a straight rootlet extending from the basal IS toward the BB, whereas canine cones displayed a curvilinear rootlet architecture (Takahashi et al., 2025). In the present dataset, canine photoreceptors showed the same subtype-dependent difference in rootlet morphology (Fig. 8A, B).

By contrast, ciliary rootlets in NHP and human retinas exhibited structural features distinct from those in canine retina. In rods, both NHP and human photoreceptors retained a generally straight rootlet architecture resembling that of canine rods, but these structures were markedly thicker than canine rod rootlets and appeared to consist of multiple filamentous elements bundled together into a single rootlet structure (Fig. 8A). Cone photoreceptors showed an even more pronounced species-dependent difference. Unlike the curvilinear rootlet architecture observed in canine cones, NHP and human cones possessed two to three thin rootlet fibers that extended from the BB in different directions. These fibers did not reach the basal end of the IS but instead terminated within the intermediate region of the IS (Fig. 8B). Thus, although the overall subtype-specific configuration was preserved across species, the detailed morphology of the rootlet differed substantially between canine and primate photoreceptors.

In contrast to the tubulin-based PSC axoneme and the aIS-like structure, no clear difference in ciliary rootlet morphology was observed between the two human donor samples (Fig. 8A, B). Thus, the species-specific features of human rootlets appeared relatively consistent across the human specimens examined. Together, these findings indicate that ciliary rootlet architecture exhibits marked rod–cone differences in all three species, while the specific morphological features of rod and cone rootlets differ substantially between canine and primate photoreceptors.

## Discussion

In this study, we used U-ExM to compare the nanoscale organization of the PSC and its associated structures across canine, NHP, and human retinas. Our findings reveal two overarching principles. First, key rod–cone differences in the tubulin-based PSC axoneme are broadly conserved across these large mammals. Second, structures surrounding the PSC, including periciliary complexes, aIS-like structures, and ciliary rootlets, exhibit substantially greater species- and subtype-dependent diversification. In this context, human photoreceptors were generally more similar to those of NHPs than to those of canines, yet also displayed additional features not readily explained by the NHP pattern alone. Together, these observations refine current concepts of mammalian photoreceptor ciliary organization by showing that conserved subtype-specific architectural principles coexist with pronounced species-specific specialization in PSC-associated compartments.

A notable implication of this comparison is that the conserved rod–cone logic of PSC architecture is implemented at different, species-specific scales. Across all three species, cones exhibited shorter CCs, longer DCs, and a larger bulge region than rods, indicating that these subtype-dependent features represent a shared architectural principle of mammalian photoreceptors rather than species-restricted specialization. At the same time, primate photoreceptors differed markedly from canine photoreceptors, with shorter CCs, more pronounced apical widening, and exceptionally long cone DCs. These observations are consistent with previous mouse–human comparisons, which reported shorter CCs in human rods than in mouse rods, as well as longer DCs in human cones relative to both human rods and mouse photoreceptors (Mercey et al., 2024). Extending beyond these comparisons, our study demonstrates that such differences are not restricted to rodent–human divergence but are also evident across large mammalian species. Moreover, through systematic subcompartment-level analysis of the PSC and comprehensive inclusion of photoreceptor subtypes, we demonstrate that interspecies and subtype-specific variations in PSC organization are more extensive and nuanced than previously appreciated. The finding that L/M-cones displayed longer DCs than S-cones across all species further suggests that PSC architecture varies not only between rods and cones, but also among cone subtypes. Although the functional basis of these features remains to be established, CC length and bulge region width likely reflect distinct structural requirements imposed by subtype- and species-specific OS organization and size.

The most conceptually important finding of this study is that the periciliary membrane is not organized uniformly across mammalian photoreceptors. In canine rods, the Whirlin-positive periciliary membrane domain was confined to the IS-facing side of the CC, whereas in primate rods and in cones of all three species it extended circumferentially around the CC. This organization implies that, in canine rods, only one side of the CC membrane is apposed to the periciliary membrane, whereas the opposite side remains relatively exposed. This configuration closely resembles the structural arrangement often inferred from electron microscopic images of mouse rods and is broadly consistent with the conventional view of rod periciliary organization (Barnes et al., 2021; May-Simera et al., 2017; Sahly et al., 2012; Spencer et al., 2019). By contrast, the circumferential Whirlin-positive domain observed in primate rods and in cones of all three species indicates that, in these photoreceptors, the CC is surrounded by the periciliary membrane and is effectively embedded within the apical inner segment compartment. This finding substantially revises the structural concept of the periciliary region by showing that the spatial relationship between the CC membrane and the periciliary membrane differs fundamentally across species and photoreceptor subtypes. Moreover, the presence of pillar-like Whirlin-positive structures along the continuous periciliary membrane, together with their species- and subtype-dependent arrangement, suggests that the organization of USH2-associated domains is more complex than previously appreciated and warrants further investigation.

These findings also refine how calyceal processes should be interpreted across species and photoreceptor subtypes. In primate rods, the combined distribution of Whirlin, PCDH15, and β-actin strongly supports the presence of well-developed periciliary membrane and calyceal processes. In contrast, canine rods showed PCDH15 localization largely restricted to the IS/OS boundary, limited PCDH15–actin association, and a unilateral periciliary membrane organization, supporting the conclusion that calyceal processes are absent or only weakly developed in this photoreceptor population. This pattern closely resembles the rod phenotype previously described in mice and suggests that limited rod calyceal specialization may extend beyond rodents (Sahly et al., 2012). However, canine cones displayed a clear calyceal process-like molecular organization, despite the lack of ultrastructural evidence by electron microscopy. This rod–cone distinction in the canine retina raises the possibility that species currently regarded as calyceal process-negative may still harbor cone-specific or rudimentary periciliary specializations that have been overlooked. Although mouse photoreceptors are generally considered to lack typical calyceal processes, the possibility of cone-restricted calyceal process-like specializations has been raised in a limited prior study, warranting targeted re-evaluation with subtype-resolved structural and molecular analyses (Yang et al., 2010).

Human photoreceptors were broadly NHP-like in their periciliary organization, although the human samples also highlighted the difficulty of generalizing from a limited number of donor eyes with variable sample conditions. Both human replicates showed circumferential Whirlin-positive arrays and robust PCDH15/actin-defined calyceal process-like structures in rods and cones, supporting the conclusion that human periciliary complexes are fundamentally primate-like. By contrast, aIS-like structures were highly variable. They were absent from both eyes in the non-macular pericentral region of donor 1, whereas donor 2 contained variable rod profiles with aIS-like extensions. We also observed aIS-like extensions in cones, which differs from a recent study that emphasized the aIS as a human rod-specific architecture. Moreover, the additional tubulin-based scaffold described in that study was not detected here, even though the same anti-β-tubulin antibody was used (Lewis et al., 2025). Thus, our data support the idea that aIS-like structures may represent a distinctive feature of human photoreceptors, but they also indicate that their prevalence, photoreceptor population specificity, and molecular definition remain unresolved and require validation in larger human cohorts.

An important consideration is retinal topography. In this study, we did not analyze the macular or foveal region in primate and human retinas, nor did we specifically examine the canine area centralis/fovea-like region. This is relevant because foveal and perifoveal cones are known to differ from more peripheral cones in density and morphology, including elongation of cone OS (Tschulakow et al., 2018; Westheimer, 2008). Similarly, the canine retina contains a specialized area centralis with a primate fovea-like cone bouquet, characterized by high cone density and elongated IS and OS despite the absence of a true foveal pit (Beltran et al., 2014). Therefore, our conclusions should be interpreted as applying primarily to the sampled superior central, non-foveal retinal regions. Future subtype-resolved U-ExM analysis of the primate fovea and canine area centralis will be needed to determine whether cones in these specialized retinal regions exhibit additional PSC, periciliary membrane, calyceal process, or aIS-like specializations.

Several limitations should be considered when interpreting these findings. Most importantly, the human donor tissues differed substantially from the canine and NHP samples in age, postmortem interval, and fixation conditions, and therefore do not constitute a strictly matched cross-species comparison. In addition, U-ExM provides nanoscale molecular information but does not directly replace electron microscopy for defining ultrastructural boundaries. Accordingly, some of our morphological interpretations, particularly for calyceal process-like and aIS-like structures, remain inference-based at the molecular level. Despite these limitations, the consistency of multiple markers across species and photoreceptor subtypes supports the robustness of the major organizational patterns identified here. Overall, our study provides a comparative nanoscale framework for mammalian photoreceptor ciliary organization and shows that the strongest diversification occurs not in the core PSC axoneme itself, but in the associated membrane and support structures that surround it. These findings deepen and substantially update current understanding of photoreceptor ciliary architecture by revealing how conserved structural principles are modified by species- and subtype-specific specialization.

## Materials and Methods

### Animals

Cryopreserved archival retinal tissues from adult dogs (*n* = 3; 24 weeks old) and cynomolgus macaques (*Macaca fascicularis*; *n* = 2; 4 years old) were used in this study. These samples were not collected specifically for the present work, but were residual archival materials from previous studies and were reused here for comparative ultrastructural analysis; details of animal husbandry and tissue collection have been described previously (Jacobson et al., 2007; Takahashi et al., 2026). All procedures conformed to the ARVO Statement for the Use of Animals in Ophthalmic and Vision Research and were approved by the Institutional Animal Care and Use Committee of the University of Pennsylvania. Details of the individual retinal samples used in this study are provided in Supplementary Table S1.

### Human donor eye tissue

Enucleated human donor eyes were obtained from the San Diego Eye Bank. The use of postmortem human eyes was exempted of ethical approval by Institutional Review Boards at the University of Pennsylvania, yet all procedures were conducted in accordance with the university safety guidelines and regulations. Retinas used in this study were derived from an 83-year-old male donor (replicate 1) and an 84-year-old female donor (replicate 2). Only donors without ocular pathology were included. All tissues were de-identified before delivery to the laboratory. Details of the donor samples are summarized in Supplementary Table S1.

### Retinal tissue processing

Procedures for canine and NHP ocular tissues were performed as described previously (Jacobson et al., 2007; Takahashi et al., 2024). The fixation regimen was standardized across all canine and NHP specimens, consisting of 4% formaldehyde for 3 h followed by 2% formaldehyde for 24 h. After cryoprotection by sequential incubation in 15% and 30% sucrose solutions in 1× phosphate-buffered saline (PBS), all formaldehyde-fixed retinal tissues were embedded in optimal cutting temperature (OCT) compound, snap-frozen in liquid nitrogen and stored at −80°C. Frozen OCT blocks were cryosectioned at 20 μm, and the sections were stored at −20°C until further use.

Human donor retinal tissues were processed under conditions that differed from those used for canine and NHP tissues. In particular, the two human donor samples were processed under distinct postmortem and fixation conditions: replicate 1 was fixed approximately 3 h after death and remained in 4% formaldehyde for 7 days, whereas replicate 2 was fixed approximately 12 h after death and remained in 4% formaldehyde for 24 h. After fixation, human tissues were cryoprotected, embedded in OCT compound, frozen and cryosectioned as described above. Details of the procedures for individual samples are summarized in Supplementary Table S1.

### Ultrastructure Expansion Microscopy (U-ExM) on retinal cryosections

Retinal cryosections were processed using a U-ExM workflow previously established for long-term cryopreserved retinal tissues (Takahashi et al., 2026; Takahashi et al., 2025). Briefly, 20-μm-thick cryosections were thawed and washed with PBS to remove OCT compound, followed by overnight incubation in an anchoring solution containing 2% acrylamide and 1.4% formaldehyde. The sections were then incubated in monomer solution composed of 23% (w/v) sodium acrylate, 10% (w/v) acrylamide, 0.1% (w/v) N,N′-methylenebisacrylamide and 1× PBS. Gelation was performed under a coverslip using monomer solution supplemented with 0.5% ammonium persulfate and 0.5% tetramethylethylenediamine. Following gelation, samples were denatured at 95°C in buffer containing 200 mM sodium dodecyl sulfate (pH = 9) and subsequently expanded in distilled water for the first-round expansion. Post-expansion immunohistochemistry, counterstaining, and second-round expansion were carried out as described previously (Takahashi et al., 2026; Takahashi et al., 2025). Reagents used for U-ExM and fluorescence labeling are listed in Supplementary Tables S2 and S3.

### Confocal imaging and image processing

Images were acquired using a STELLARIS 8 confocal microscope (Leica Microsystems, Wetzlar, Germany) equipped with a 63× water-immersion objective (NA 1.20), as described previously (Takahashi et al., 2025). For lateral views, z-stacks were acquired at 0.4 μm intervals with an *x,y* pixel size of 45.09 nm and a scan speed of 400 Hz. Axial-view images were acquired with an *x,y* pixel size of 22.55 nm and a scan speed of 100 or 200 Hz. Representative and quantitative images were processed in Leica Application Suite X (LAS X; Leica Microsystems) for LIGHTNING deconvolution with default parameters, as well as for generation of merged images and maximum-intensity projections (MIPs). Three-dimensional (3D) rendering was performed using Imaris software (version 10.2.0; Oxford Instruments, Bristol, UK) in blend mode.

### Quantification

#### Expansion factor (EF) calculation

The EF was estimated by dividing the width of the acetylated α-tubulin (AcTub)-labeled basal body (BB) axoneme in U-ExM-processed canine and NHP samples by the reported width of the non-expanded centriole in human U2OS cells (231.3 nm), assuming conservation of BB (mother centriole) width across mammalian species (Faber et al., 2023; Mercey et al., 2024; Mercey et al., 2022). Because BB axoneme width was comparable between canine and NHP retinas, a single EF of 3.64 was determined by averaging the values obtained from canine and NHP photoreceptors. This EF was applied to all quantitative measurements.

#### Protein signal length, width, and distance

Protein signal lengths, widths and distances from the proximal end of the BB were measured manually in the corresponding regions using the “Draw scalebar” function in LAS X software, as described previously (Takahashi et al., 2025). All measurements were subsequently corrected using the EF.

#### Line-profile analysis

Intensity profiles were extracted using the “Quantify” tool in LAS X. The exported intensity and distance values were imported into Microsoft Excel, and distance values were corrected by the EF. Intensities were normalized for each channel such that the maximum value within the measured range was set to 1. Line-profile plots were generated in Microsoft Excel.

### Statistical analysis

Pairwise comparisons were performed using Welch’s *t*-test, whereas comparisons involving more than two groups were analyzed using Welch’s one-way ANOVA followed by Dunnett’s T3 multiple-comparison test. Quantitative data are presented as scatter dot plots, with central lines and error bars indicating the mean and standard deviation (± SD), respectively. Statistical significance was defined as follows: ns, not significant (*P* > 0.05); *P < 0.05, **P < 0.01, ***P < 0.001, and ****P < 0.0001. All statistical analyses were performed using Prism 10 (GraphPad, Boston, MA, USA). Source data for all graphs are provided in the Supplementary Data.

## Supplementary Material

Supplement 1 **Supplementary figure S1. Comparison of basal body width for determination of the expansion factor. (A)** Representative confocal images of photoreceptor sensory cilium (PSC) architecture in rod and cone photoreceptors from canine and non-human primate (NHP) retinas. PSC architecture and photoreceptor somata were visualized by acetylated α-tubulin (AcTub) immunolabeling (magenta) and N-hydroxysuccinimide (NHS) -ester pan-staining (gray), respectively. All images are shown as maximum intensity projections (MIPs). Scale bars, 1 μm, shown without correction for the expansion factor. **(B)** Schematic diagram illustrating basal body (BB) width measurement. **(C)** Cross-species comparison of BB width between rods and cones. Central lines indicate the mean, and error bars represent ± SD. No significant differences were observed among any pairwise comparisons (Welch’s one-way ANOVA followed by Dunnett’s T3 multiple-comparison test; n.s., *P* > 0.05).

Supplement 2 **Supplementary figure S2. Comparison of CC length between central and peripheral regions in the canine retina. (A, B)** Confocal images showing the length of the connecting cilium (CC) in the central and peripheral regions of the canine retina for rods (A) and cones (B). The tubulin axoneme and the CC were visualized by AcTub/CEP290 immunolabeling (magenta/green), and the photoreceptor soma was visualized by NHS-ester pan-staining (gray). All images are shown as MIPs. Scale bars, 1 μm, corrected for the expansion factor. **(C, D)** Quantification of CC length in the central and peripheral retina for rods (C) and cones (D). Central lines indicate the mean, and error bars represent ± SD. ****P < 0.0001, as assessed by Welch’s *t*-test.

Supplement 3 **Supplementary figure S3. Representative molecular markers of the CC.** Representative confocal images showing additional molecular markers of tubulin-based PSC structures and CC length in rod and cone photoreceptors from canine, NHP, and human retinas. Upper panels show AcTub (magenta) and SPATA7 (green) immunolabeling, whereas lower panels show glutamylation (green) labeling of the PSC. Consistent with the results shown in Figs. 1 and 3, NHP and human photoreceptors have shorter CCs than canine photoreceptors, whereas the difference in CC length between rods and cones is maintained across all species. In addition, NHP and human cone photoreceptors possess markedly longer DCs than those in their rod counterparts and in canine photoreceptors. All images are shown as MIPs. Scale bars, 1 μm, corrected for the expansion factor.

Supplement 4 **Supplementary figure S4. Comparison of CC and DC length between S-cones and L/M-cones. (A)** Confocal images showing PSC structures in canine, NHP, and human retinas for S-cones (blue) and L/M-cones (red). The tubulin axoneme and CC were visualized by glutamylation immunolabeling (green). All images are shown as MIPs. Scale bars, 1 μm, corrected for the expansion factor. **(B, C)** Quantification of CC length (B) and DC length (C) in the three species (canine, circles; NHP, squares; human, triangles). Central lines indicate the mean, and error bars represent the ± SD. Yellow dashed lines indicate the mean values for rod photoreceptors of each species, derived from the corresponding measurements shown in Fig. 1E, J and Fig. 3C, E. Data were obtained from two or three individual eyes for each species. **P < 0.01, ****P < 0.0001, as assessed by Welch’s *t*-test.

Supplement 5 **Supplementary figure 5. Ectopic localization of Whirlin in canine cone photoreceptors.** Representative confocal images showing the localization of whirlin (magenta) and PCDH15 (green) in periciliary structures of canine cone photoreceptors from three independent biological replicates. Upper panels show merged images of whirlin, PCDH15, and NHS-ester counterstaining (gray), whereas lower panels show whirlin and PCDH15 labeling only. Yellow arrowheads indicate ectopic localization of whirlin. All images are shown as 3D-rendered images. Scale bars, 1 μm, corrected for the expansion factor.

Supplement 6 **Supplementary Table S1:** List of archival retinas used in this study.
**Supplementary Table S2:** Reagents and materials used for tissue processing and U-ExM.
**Supplementary Table S3:** Antibodies and regents used for immunolabeling.

Supplement 7 **Supplementary Data:** Source data underlying the quantitative analyses.

## Data Availability

All numerical source data underlying the quantitative analyses are included in the Supplementary Data. Representative images are shown in the main figures and supplementary figures. Source images are available from the corresponding authors upon reasonable request.
