# Cross-species molecular mapping of the photoreceptor sensory cilium and periciliary structures identifies conserved and species-specific architectural features

**DOI:** 10.64898/2026.05.24.727487

**Published:** 2026-08-04

**Authors:** Kei Takahashi, Mayuna Obayashi, Raghavi Sudharsan, William A. Beltran

## Abstract

Photoreceptor sensory cilia comprise a microtubule-based structural core and the phototransductive outer segment. The microtubule-based ciliary axoneme extends from the basal body through the connecting cilium into the outer segment, thereby linking the inner and outer segments. Around this central axis, specialized periciliary structures are distributed across the apical inner segment and basal outer segment. Yet how these ciliary and periciliary structures differ between rods and cones and across mammals remains incompletely defined. Using ultrastructure expansion microscopy, we compared their nanoscale molecular organization across canine, non-human primate (NHP), and human retinas. Key rod–cone differences in ciliary organization were broadly conserved among all three species, whereas the scale and morphology of individual ciliary subcompartments differed between species. Periciliary structures showed even greater species-specific diversification, particularly in rods. Human photoreceptors shared several structural features with NHP photoreceptors but also displayed inter-individual variability in accessory inner segment-like structure. These findings refine current understanding of photoreceptor sensory cilia and associated periciliary structures in large mammals, revealing that conserved rod–cone differences coexist with pronounced species-specific structural and molecular specializations.

## Full Text Availability

The license terms selected by the author(s) for this preprint version do not permit archiving in PMC. The full text is available from the preprint server.

