# Supplementary figures and images for: Cross-species molecular mapping of the photoreceptor sensory cilium and periciliary complexes identifies conserved and species-specific architectural features
Source: bioRxiv. 2026 May 27:2026.05.24.727487. Preprint. [Version 1] doi: 10.64898/2026.05.24.727487 (PMC13232334; doi:10.64898/2026.05.24.727487)

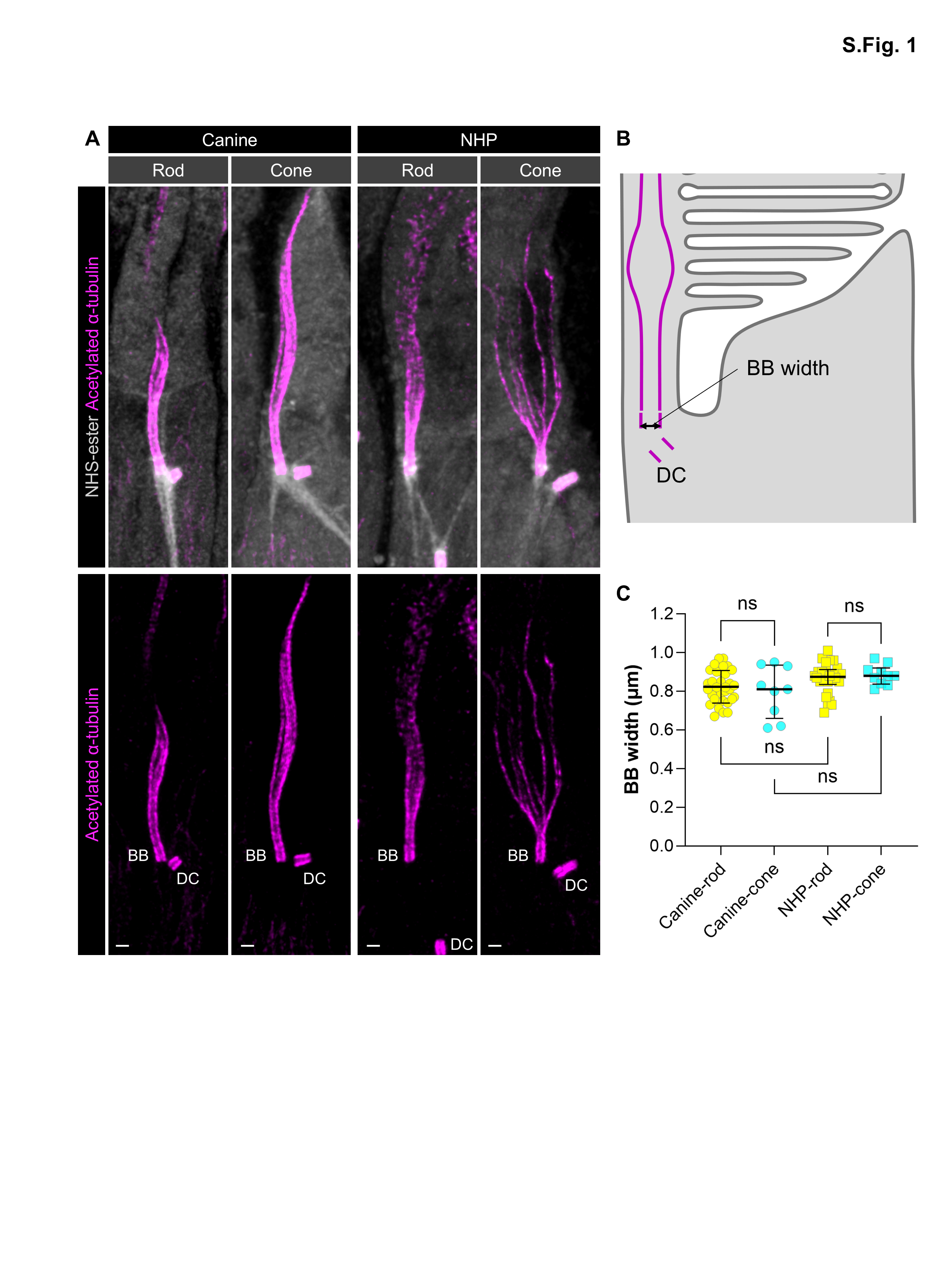

Supplement: Supplement 1 — Supplementary figure S1. Comparison of basal body width for determination of the expansion factor. (A) Representative confocal images of photoreceptor sensory cilium (PSC) architecture in rod and cone photoreceptors from canine and non-human primate (NHP) retinas. PSC architecture and photoreceptor somata were visualized by acetylated α-tubulin (AcTub) immunolabeling (magenta) and N-hydroxysuccinimide (NHS) -ester pan-staining (gray), respectively. All images are shown as maximum intensity projections (MIPs). Scale bars, 1 μm, shown without correction for the expansion factor. (B) Schematic diagram illustrating basal body (BB) width measurement. (C) Cross-species comparison of BB width between rods and cones. Central lines indicate the mean, and error bars represent ± SD. No significant differences were observed among any pairwise comparisons (Welch’s one-way ANOVA followed by Dunnett’s T3 multiple-comparison test; n.s., P > 0.05). [file media-1.tif]

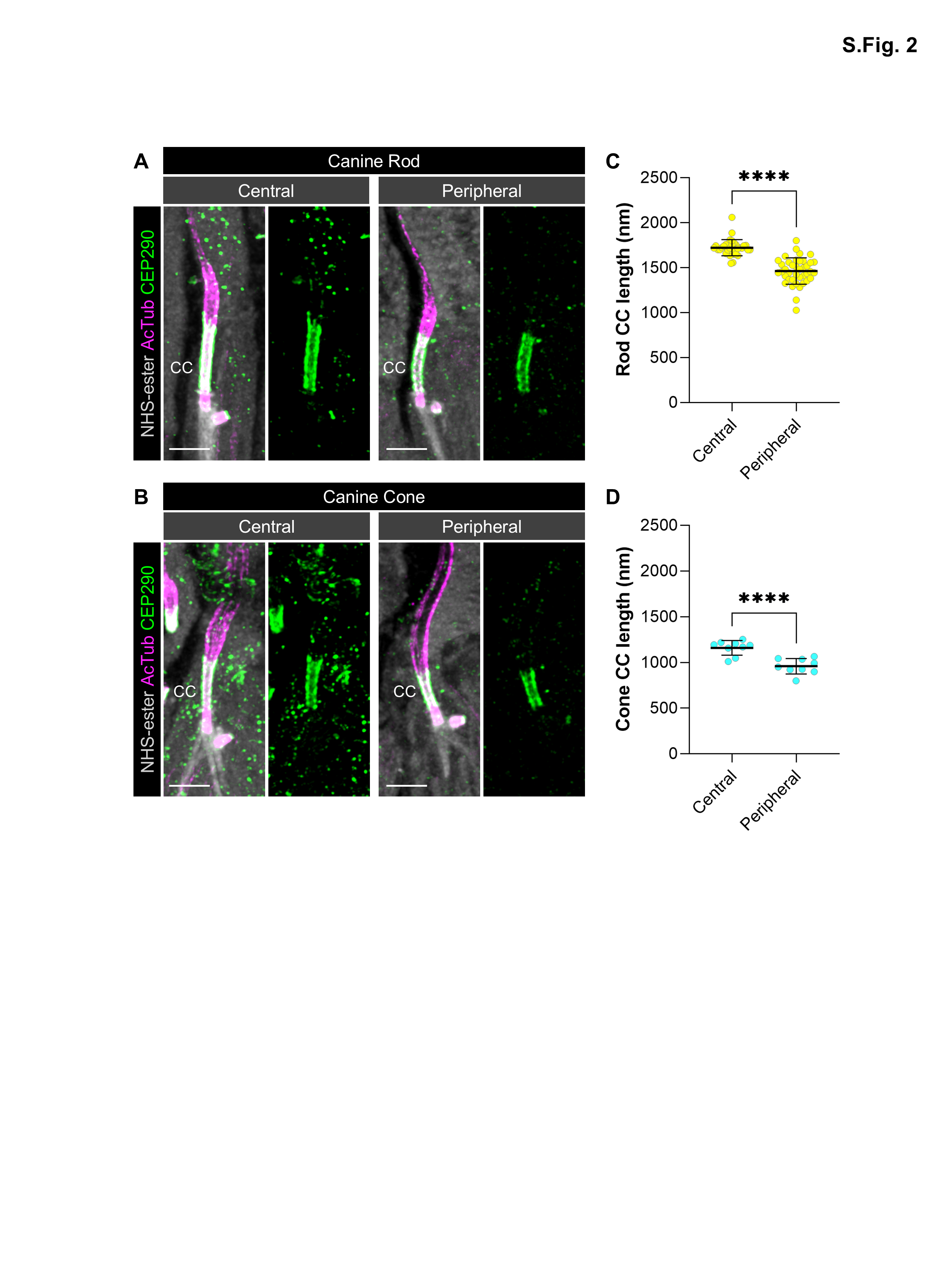

Supplement: Supplement 2 — Supplementary figure S2. Comparison of CC length between central and peripheral regions in the canine retina. (A, B) Confocal images showing the length of the connecting cilium (CC) in the central and peripheral regions of the canine retina for rods (A) and cones (B). The tubulin axoneme and the CC were visualized by AcTub/CEP290 immunolabeling (magenta/green), and the photoreceptor soma was visualized by NHS-ester pan-staining (gray). All images are shown as MIPs. Scale bars, 1 μm, corrected for the expansion factor. (C, D) Quantification of CC length in the central and peripheral retina for rods (C) and cones (D). Central lines indicate the mean, and error bars represent ± SD. ****P < 0.0001, as assessed by Welch’s t-test. [file media-2.tif]

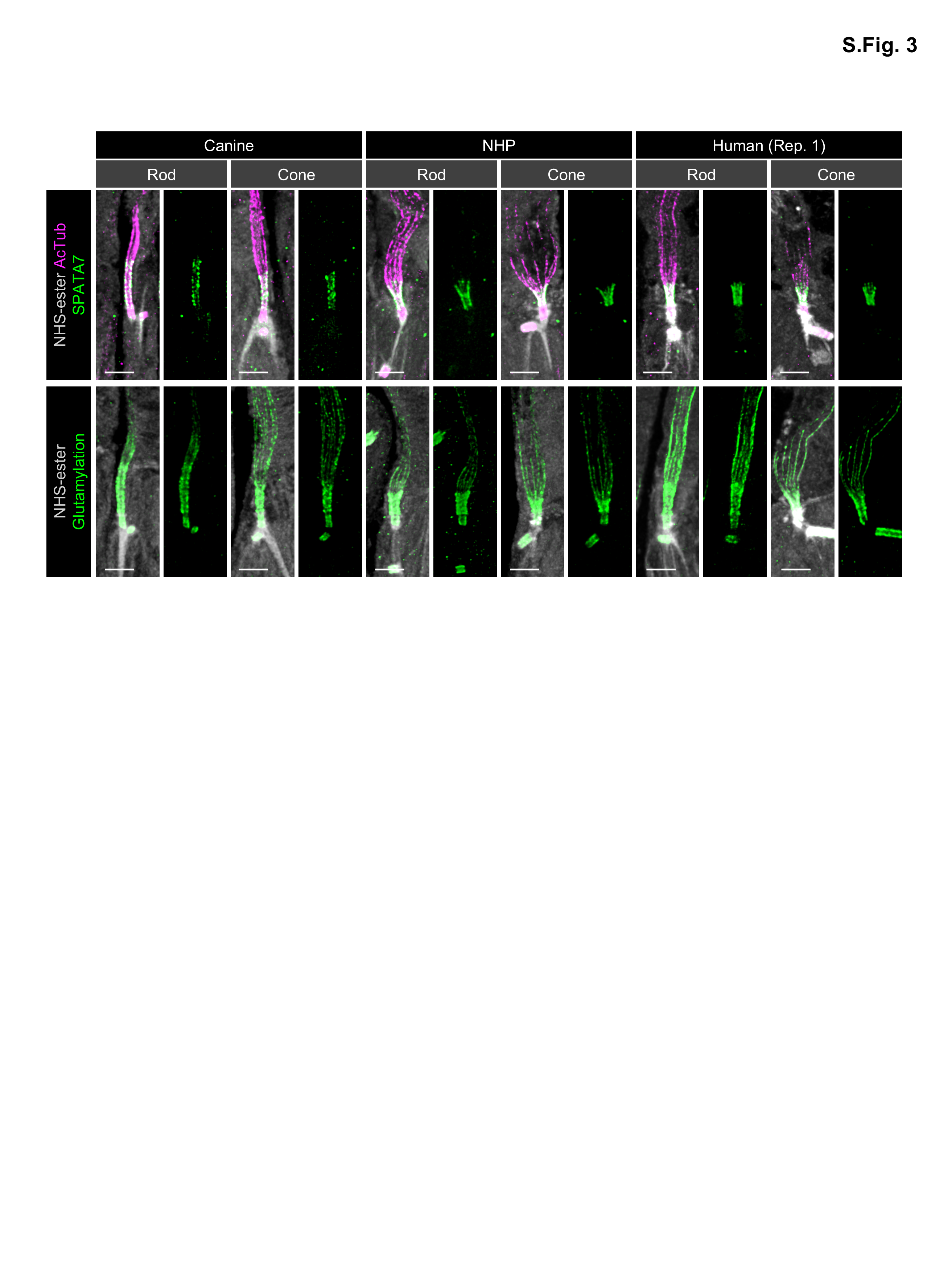

Supplement: Supplement 3 — Supplementary figure S3. Representative molecular markers of the CC. Representative confocal images showing additional molecular markers of tubulin-based PSC structures and CC length in rod and cone photoreceptors from canine, NHP, and human retinas. Upper panels show AcTub (magenta) and SPATA7 (green) immunolabeling, whereas lower panels show glutamylation (green) labeling of the PSC. Consistent with the results shown in Figs. 1 and 3, NHP and human photoreceptors have shorter CCs than canine photoreceptors, whereas the difference in CC length between rods and cones is maintained across all species. In addition, NHP and human cone photoreceptors possess markedly longer DCs than those in their rod counterparts and in canine photoreceptors. All images are shown as MIPs. Scale bars, 1 μm, corrected for the expansion factor. [file media-3.tif]

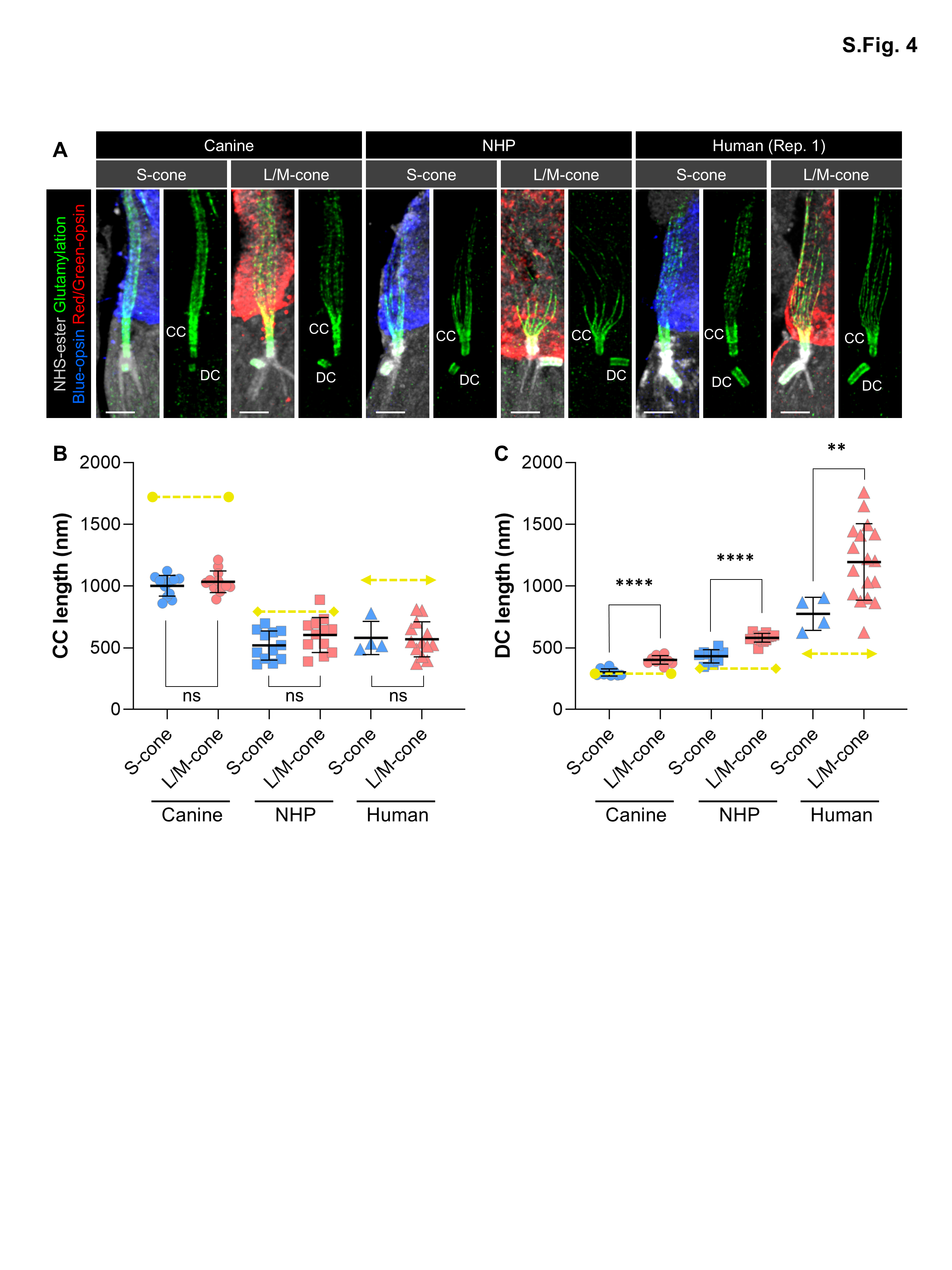

Supplement: Supplement 4 — Supplementary figure S4. Comparison of CC and DC length between S-cones and L/M-cones. (A) Confocal images showing PSC structures in canine, NHP, and human retinas for S-cones (blue) and L/M-cones (red). The tubulin axoneme and CC were visualized by glutamylation immunolabeling (green). All images are shown as MIPs. Scale bars, 1 μm, corrected for the expansion factor. (B, C) Quantification of CC length (B) and DC length (C) in the three species (canine, circles; NHP, squares; human, triangles). Central lines indicate the mean, and error bars represent the ± SD. Yellow dashed lines indicate the mean values for rod photoreceptors of each species, derived from the corresponding measurements shown in Fig. 1E, J and Fig. 3C, E. Data were obtained from two or three individual eyes for each species. **P < 0.01, ****P < 0.0001, as assessed by Welch’s t-test. [file media-4.tif]

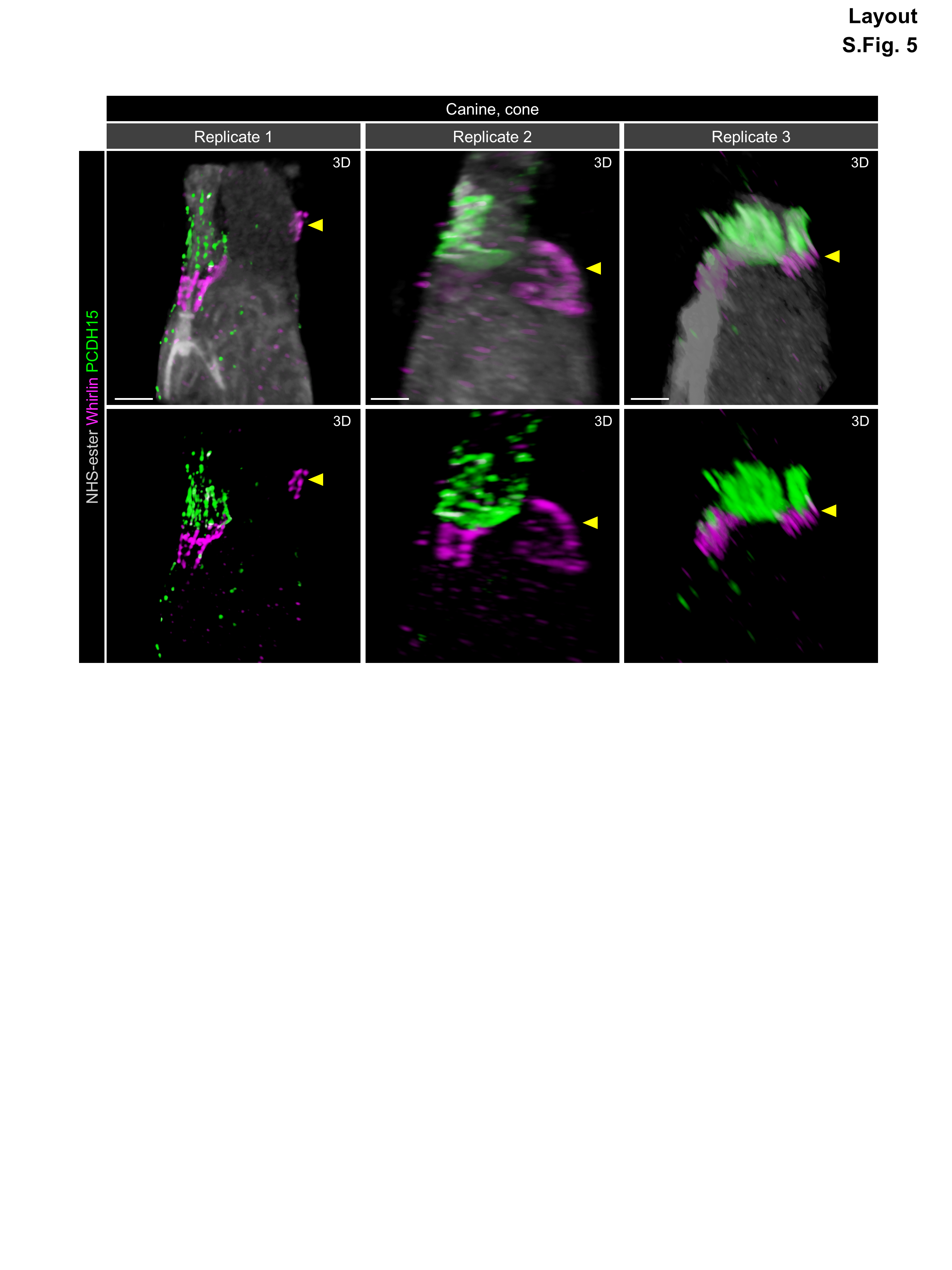

Supplement: Supplement 5 — Supplementary figure 5. Ectopic localization of Whirlin in canine cone photoreceptors. Representative confocal images showing the localization of whirlin (magenta) and PCDH15 (green) in periciliary structures of canine cone photoreceptors from three independent biological replicates. Upper panels show merged images of whirlin, PCDH15, and NHS-ester counterstaining (gray), whereas lower panels show whirlin and PCDH15 labeling only. Yellow arrowheads indicate ectopic localization of whirlin. All images are shown as 3D-rendered images. Scale bars, 1 μm, corrected for the expansion factor. [file media-5.tif]
