# Supplementary material for: Cross-species molecular mapping of the photoreceptor sensory cilium and periciliary complexes identifies conserved and species-specific architectural features
Source: bioRxiv. 2026 May 27:2026.05.24.727487. Preprint. [Version 1] doi: 10.64898/2026.05.24.727487 (PMC13232334; doi:10.64898/2026.05.24.727487)
Supplement: Supplement 6 — Supplementary Table S1: List of archival retinas used in this study. Supplementary Table S2: Reagents and materials used for tissue processing and U-ExM. Supplementary Table S3: Antibodies and regents used for immunolabeling. [file media-6.pdf]

Supplementary Table S1: List of archival retinas used in this study.

Species	ID	Sex	Age	Eye	Postmortem interval / Fixation conditions	Ref.
Canine	RC702	M	24 weeks	OS	< 5 mins / 4% FA 3 hrs, 2% FA 24 hrs	Takahashi et al., 2026
	RC703	M	24 weeks	OS		
	RC706	F	24 weeks	OS		
NHP (cynomolgus macaques)	21946	F	4 years	OD	< 20 mins / 4% FA 3 hrs, 2% FA 24 hrs	Jacobson et al., 2007
	21961	F	4 years	OS		
Human	125546 ODWE (replicate 1)	M	83 years	OS, OD	~ 3 hrs / 4% FA 7 days	NA
	123991 ODWE (replicate 2)	F	84 years	OS	~ 12 hrs / 4% FA 24 hrs	NA

F, female; FA, formaldehyde; M, male; NHP, non-human primate; OD, right eye; OS, left eye.

Supplementary Table S2. Reagents and materials used for tissue processing and U-ExM.

Product	Supplier	Cat#
**Tissue processing and storage**		
20% paraformaldehyde solution	Electron microscopy sciences	15713-S
Sucrose	Fiser chemical	S2-500
Tissue-Tek O.C.T. Compound	Sakura finetek	4583
Tissue Embedding Disposable Molds	EBSciences	H1513
**U-ExM**		
14-mm microwell/35-mm petri dish	MatTek	P35G-1.5-14-C
12 mm Circular Cover Glasses	Fisher Scientific	12541001
Acrylamide (AA)	Sigma-Aldrich	A4058
Ammonium Persulfate (APS)	Bio-Rad	1610700
Formaldehyde solution	Sigma-Aldrich	F8775
ImmEdge Pen	Vector laboratories	H-4000
Nuclease-Free Water	Invitrogen	AM9937
N, N′-methylenbisacrylamide (BIS)	Sigma-Aldrich	M1533
Phosphate Buffered Saline (PBS), 10x	Bio-Rad	161-0780
Poly-D-Lysine	Gibco	A3890401
Sodium Acrylate (SA)	Sigma-Aldrich	408220
Sodium Chloride (NaCl)	Fisher Chemical	S271-3
Sodium Dodecyl Sulfate (SDS)	Fisher Chemical	BP166-500
Tetramethylethylenediamine (TEMED)	Bio-Rad	161-0800
Tris Base	Fisher Chemical	BP152-5

Supplementary Table S3: Antibodies and regents used for immunolabeling.

Primary antibodies

Antibody	Host Organism	Clonality / Isotype	Cat# or Ref.	Dilution	RRID
Acetylated α-tubulin	Mouse	Monoclonal IgG2b	T7451	1/1000	AB_609894
Acetylated α-tubulin	Rabbit	Monoclonal IgG	ab179484	1/1000	AB_2890906
β-actin	Rabbit	Polyclonal IgG	ab8227	1/200	AB_2305186
Blue opsin	Rabbit	Polyclonal IgG	AB5407	1/500	AB_177457
β-tubulin	Mouse	Monoclonal IgG1	T4026	1/500	AB_477577
CEP164	Rabbit	Polyclonal IgG	22227-1-AP	1/200	AB_2651175
CEP290	Rabbit	Polyclonal IgG	22490-1-AP	1/200	AB_10973679
Cytochrome C	Sheep	Polyclonal IgG	C9616	1:200	AB_532232
Glutamylation (GT335)	Mouse	Monoclonal IgG1	AG-20B-0020	1/1000	AB_2490210
LCA5 (lebercilin)	Rabbit	Polyclonal IgG	19333-1-AP	1/200	AB_2878576
PCDH15	Sheep	Polyclonal IgG	AF6729	1/300	AB_10892338
POC5	Rabbit	Polyclonal IgG	A303-341A	1/200	AB_10971172
Red/Green opsin	Rabbit	Polyclonal IgG	AB5405	1:300	AB_177456
Rhodopsin	Rabbit	Polyclonal IgG	AB9279	1/1000	AB_11210489
Rootletin	Human	Monoclonal IgG	HCA009	1/200	AB_2085504
Rootletin	Mouse	Monoclonal IgG1	sc-374056	1/200	AB_10918081
RP1	Chicken	Polyclonal IgY	Custom (Liu et al., 2002)	1/300	NA
SPATA7	Rabbit	Polyclonal IgG	12020-1-AP	1/200	AB_2195380
Whirlin	Rabbit	Polyclonal IgG	25881-1-AP	1/200	AB_2880280

Secondary antibodies

Target	Host Organism	Clonality / Isotype	Fluorescent	Cat#	Dilution	RRID
Chicken IgY (H+L)	Goat	Polyclonal IgG	Alexa Fluor 488	A11039	1:1000	AB_2534096
Mouse IgG_1	Goat	Polyclonal IgG	Alexa Fluor 488	A21121	1:1000	AB_2535764
Mouse IgG_{2b}	Goat	Polyclonal IgG	Alexa Fluor 488	A21141	1:1000	AB_2535778
Sheep IgG (H+L)	Donkey	Polyclonal IgG	Alexa Fluor 488	A11015	1:1000	AB_2534082
Mouse IgG_1	Goat	Polyclonal IgG	Alexa Fluor 568	A21124	1:1000	AB_2535766
Mouse IgG (H+L)	Donkey	Polyclonal IgG	Alexa Fluor 568	A10037	1:1000	AB_11180865
Rabbit IgG (H+L)	Goat	Polyclonal IgG	Alexa Fluor 568	A11036	1:1000	AB_10563566
Rabbit IgG (H+L)	Donkey	Polyclonal IgG	Alexa Fluor 568	A10042	1:1000	AB_2534017

Fluorescent conjugates

Product	Fluorescent	Supplier	Cat#	Stock solution	Dilution
Hoechst 33342	Hoechst 33342	Invitrogen	62249	20 mM	1:5000
NHS-ester	Atto 647N	Sigma-Aldrich	18373	2 mg/mL	10 µg/mL

RRID, research resource identifiers.
